# Home-based transcranial direct current stimulation for persistent pain state in rheumatoid arthritis: a randomized trial

**DOI:** 10.1186/s42358-026-00535-1

**Published:** 2026-05-20

**Authors:** Stephanie Pilotti, Lucas Denardi Dória, Maria Luisa Gasparini, Natália Garcia dos Santos, Barbara Regina França, André Luiz Silveira Mallmann, André Lucas Ribeiro, Leonardo Peterson dos Santos, Daniel Nóbrega de Moraes, Rafaela Cavalheiro do Espírito Santo, Claiton Viegas Brenol, Iraci L. S. Torres, Wolnei Caumo, Ricardo Machado Xavier

**Affiliations:** 1 Programa de Pós-Graduação em Ciências Médicas Faculdade de Medicina, Universidade Federal do Rio Grande do Sul, Porto Alegre, RS Brazil; 2 Laboratório de Doenças Autoimunes, Serviço de Reumatologia, Hospital de Clínicas de Porto Alegre, Porto Alegre, RS Brazil; 3 Laboratório de Dor e Neuromodulação, Hospital de Clínicas de Porto Alegre, Porto Alegre, RS Brazil; 4 Health Research and Innovation Science Centre, Faculty of Health Sciences, Klaipeda University, Klaipeda, Lithuania; 5 Department: Laboratory of Autoimmune Diseases, Institution: Hospital de Clínicas de Porto Alegre at UFRGS, Ramiro Barcelos, 2350 - Bairro Rio Branco, Porto Alegre, RS CEP 90035-003 Brazil

**Keywords:** Rheumatoid arthritis, Transcranial direct current stimulation (tDCS), Chronic pain, Central sensitization

## Abstract

**Background:**

Although effectively controlling inflammation, up to 50% of patients with rheumatoid arthritis (RA) experience persistent pain, associated with central sensitization and neuroinflammation. Home-based transcranial direct current stimulation (tDCS) has shown efficacy in chronic pain.

**Objective:**

To investigate whether anodal tDCS (a-tDCS) is more effective than sham stimulation in reducing pain.

**Methods:**

Randomized, double-blind, sham-controlled trial with 34 women (18–70 years) with RA and VAS > 40 mm. Participants were randomized to receive a-tDCS (*n* = 17) or sham tDCS (*n* = 17). Home-based tDCS (2 mA, 20 min/day) or sham (2 mA, 90 s) for four weeks, using anodal-left M1 montage. Primary outcomes was pain (Visual Analogue Scale, VAS), Secondary outcomes included pressure pain threshold (PPT), central sensitization (CSI), physical function (HAQ-DI), fatigue (FACIT-F), CNS biomarkers, adherence, and safety.

**Results:**

Mean VAS reduction from baseline was greater in the a-tDCS group (-33.5 mm) versus s-tDCS (-14.1 mm), with a between-group difference of -19.4 mm (95% CI, -29.3 to -9.5; *p* = 0.003). Linear mixed-effects models showed that a-tDCS reduced VAS pain by 27.7% versus 6.0% with sham, a between-group difference of 21.7% (Cohen’s d = 1.15). HAQ-DI improved by 38.0% versus 7.2% (ES = 1.10). a-tDCS reduced analgesic use by 62% (RR = 0.38; 95% CI, 0.18–0.79). Exploratory analyses suggested that neuroplasticity mechanisms might mediate these effects.

**Conclusion:**

Home-based a-tDCS effectively reduced pain, disability, and analgesic use in RA patients with persistent pain without objective inflammation.

**Supplementary Information:**

The online version contains supplementary material available at 10.1186/s42358-026-00535-1.

## Introduction

Rheumatoid arthritis (RA) is a chronic autoimmune disease characterized by systemic inflammation primarily affecting the joints and often accompanied by persistent pain, even in the absence of active disease [1, 2]. This pain may persist despite adequate inflammatory control with disease-modifying antirheumatic drugs (DMARDs) or biologic therapy, affecting 30–50% of patients in clinical remission [3, 4]. Such persistent pain is often discordant with disease activity scores and laboratory markers, reflecting central sensitization mechanisms beyond peripheral inflammation. This process, characterized by neuroinflammation, glutamate-mediated excitatory signaling, and impaired descending inhibition, increases synaptic excitability and reduces inhibitory control within nociceptive pathways, maintaining a state of heightened pain sensitivity [5].

Despite the proven efficacy of DMARDs in controlling inflammatory activity, pain often persists in patients with rheumatoid arthritis during remission, even after optimized pharmacological management with analgesics, nonsteroidal anti-inflammatory drugs (NSAIDs), opioids, antidepressants, anticonvulsants, and corticosteroids [2, 6]. This therapeutic refractoriness underscores the need for evidence-based non-pharmacological interventions targeting central mechanisms of pain modulation Among non-pharmacological approaches, transcranial direct current stimulation (tDCS) has emerged as a promising neuromodulatory strategy [7–9]. This noninvasive technique delivers low-intensity electrical current (1–2 mA) through scalp electrodes to modulate cortical excitability and neuroplasticity [10–12]. Anodal stimulation enhances cortical excitability, while cathodal stimulation reduces it, restoring excitatory–inhibitory balance and strengthening descending pain inhibition.

The effects of tDCS on pain and cognition have been associated with baseline neuroplasticity, indirectly indexed by serum levels of brain-derived neurotrophic factor (BDNF) [13, 14]. Evidence from meta-analyses suggests that stimulation of the primary motor cortex (M1) may provide slightly superior analgesic effects compared to stimulation of the dorsolateral prefrontal cortex (DLPFC) [7, 9], likely due to its more direct modulation of sensory-discriminative pain processing pathways and thalamocortical circuits [7, 9]. Consistent benefits in neuropathic pain further support the role of M1-tDCS in restoring cortical excitability balance within pain networks. Although these findings support the use of tDCS in several chronic pain disorders, evidence remains scarce in inflammatory diseases such as RA, particularly in patients experiencing persistent pain during remission—a population in which peripheral inflammation is controlled, but central pain mechanisms likely predominate. Investigating the efficacy of tDCS in this context could help bridge this gap, offering a mechanistically grounded, non-pharmacological strategy to complement conventional therapy.

Therefore, this study aimed to determine whether anodal-(a)-tDCS) applied over the M1 is more effective than sham stimulation in reducing pain intensity in patients with rheumatoid arthritis and persistent pain during low inflammatory activity. Secondary objectives were to assess the effects of a-tDCS on improving functional capacity, analgesic use, pain-related disability, pain pressure threshold (PPT), fatigue (FACIT), and treatment adherence. Exploratory analyses also examined biological and psychophysical predictors of treatment response, including changes in serum brain-derived neurotrophic factor (BDNF) levels and central sensitization scores.

## Methods: Participants, interventions, and outcomes

### Study design and participants eligibility

This is a randomized, double-blind, sham-controlled clinical trial, conducted at the Hospital de Clínicas de Porto Alegre (HCPA), Brazil and approved by the Research Ethics Committee (IRB, 36972320.6.0000.5327). Participants provided written and oral consent, and the trial is registered under number 2024–0014. The study was performed at the Rheumatology Division of HCPA between January 2023 and December 2024. In addition, the study was registered in the Brazilian Registry of Clinical Trials (ReBEC) (registered under number RBR-9tnwp85). The trial protocol follows CONSORT guidelines [15]. For intervention and primary outcome, data is available under request (rmaxavier@hcpa.edu.br).

### Eligibility criteria

The study involved 34 right-handed, literate adult females between the ages of 18 and 70 with rheumatoid arthritis (RA) with low objective findings of inflammation and persistent pain (≥ 3 months). It included patients from the RA outpatient clinic at HCPA, diagnosed per 2010 ACR/EULAR criteria [16]; generalized pain > 3 months, non-mechanical/inflammatory, assessed by a certified rheumatologist; (3) pain > 4 cm on the visual analogue scale (VAS), reported by the participant in consultations within 3 months prior to inclusion; swollen joint count ≤ 1; C-reactive protein (CRP) < 10 mg/L and erythrocyte sedimentation rate (ESR) < 20 mm/h in recent tests within 3 months of inclusion; unchanged DMARD regimen for 6 months prior to the study, and prednisone ≤ 5 mg/day during the same period; stable antidepressant or anticonvulsant doses with no changes in the 30 days prior; Exclusion criteria followed established guidelines for tDCS (i.e. history of neurosurgery, traumatic brain injury, history of neurological disease or stroke; intracranial metallic implants) [17] and uncompensated clinical conditions (e.g., heart, kidney, liver disease); cancer, and illicit drug use within the past six months.

### Randomization and blinding

Participants were randomly assigned, in a 1:1 ratio, to receive either active tDCS (a-tDCS) or sham tDCS (s-tDCS). Randomization was performed using a computer-generated sequence created on Randomization.com. Two independent investigators conducted the randomization procedure before recruitment, and the allocation codes were placed in sealed, opaque envelopes. Only the engineer responsible for device programming immediately before the first stimulation session opened these envelopes. The study ensured blinding, with both participants and research staff unaware of treatment allocation. The engineer programmed the tDCS device according to the randomization, maintaining blinding throughout the study.

### Intervention

**Fig. 1:**
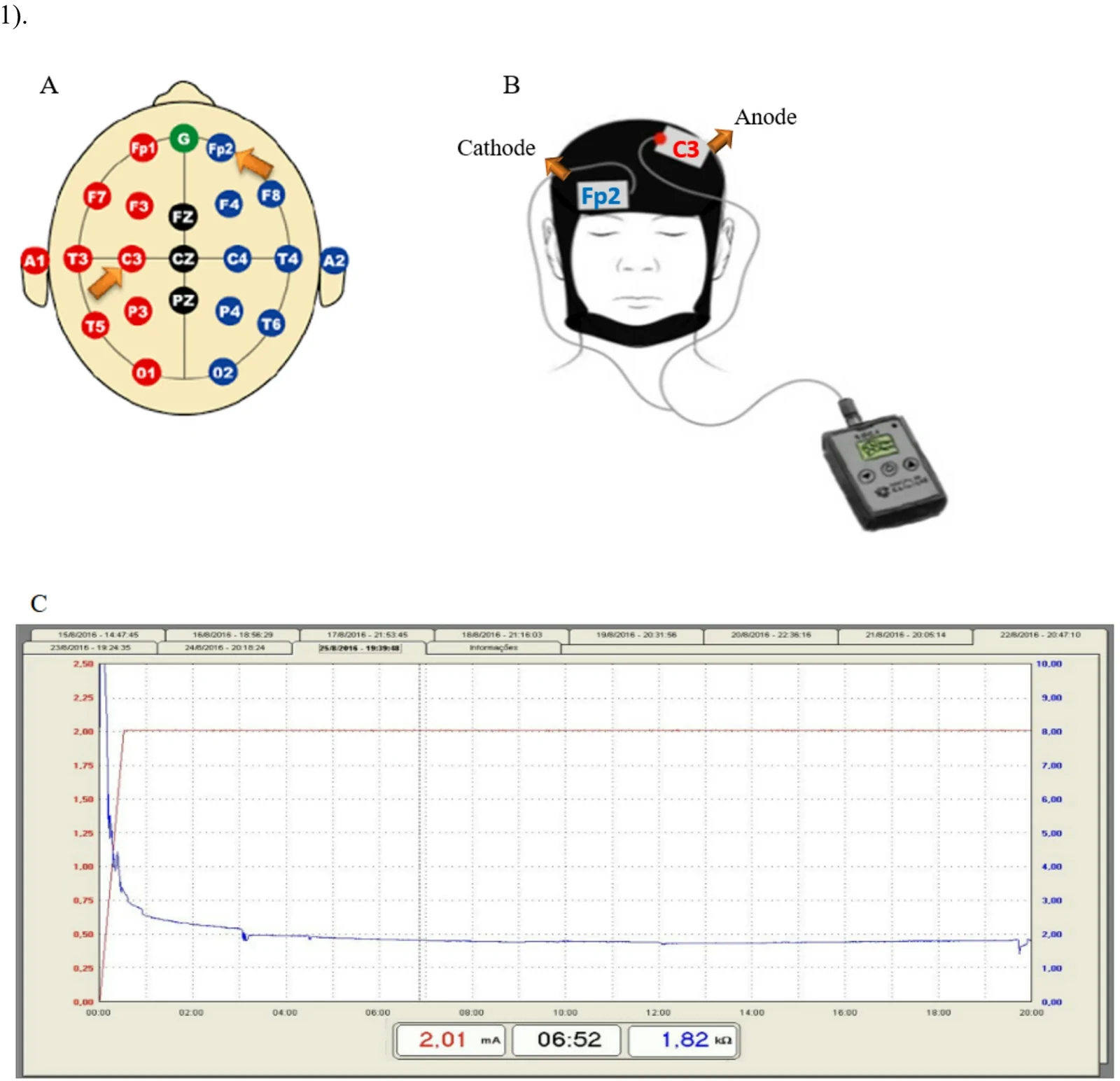
(**A**) Electrode placement according to the International 10–20 EEG System [20]. (**B**) Arrows indicate electrode positions at Fp2 (right supraorbital area) and C3 (left primary motor cortex). (**C**) Active tDCS stimulation setup. (**D**) Sham tDCS stimulation setup

We used a HB-tDCS headset, developed and validated with HCPA’s Biomedical Engineering department and registered with ANVISA (Nº80079190028) [14, 18–20]. The device monitors contact impedance and session data, halting if impedance exceeds 1 mA for over 5 s or current fluctuates by more than 10%. The tDCS was administered using two conductive rubber electrodes. Electrodes (35 cm²) were moistened with saline and positioned using a neoprene cap. The electrodes were placed with the anode over C3 (left-M1) and the cathode over Fp2 (right supraorbital), based on the international 10–20 electroencephalography system (Figure 1). The treatment was administered over five consecutive days, spanning a period of four weeks, for 20 sessions. Participants received the programmed device for home use, along with a single in-person training session. A biomedical engineer programmed the device to operate for a minimum of 16 h between sessions and to provide automatic control for both active and sham conditions. During this session, the researcher provided detailed instructions regarding home usage, care, and storage of the device throughout the treatment period. For the a-tDCS, the current applied was 2 mA for 20 min [21]. For s-tDCS conditions, the anode and cathode positions were the same as those for a-tDCS. The sham stimulation protocol consisted of brief stimulation periods applied at three time points during the session: at the beginning, at minute 10, and at minute 20. At each time point, the current was ramped up from 0 to 2 mA over 20 s, maintained for 30 s, and then ramped down over 20 s. No stimulation was delivered between these periods. This procedure was implemented to mimic the transient sensory effects of active stimulation and maintain participant blinding (details in Supplemental 1).

## Experimental design

The evaluations outcomes were conducted on 3 visits. First visit: Upon arriving at the hospital, patients provided their written, formal consent; diagnosis was confirmed, and baseline assessment procedures were completed. The first 20 min treatment session was administered, which also included a training session on how to use the device at home. Second visit: The follow-up questionnaires will be administered remotely via telephone call. Third visit: The patient returned the device to the lab after completing the assessment at the end of the treatment, which took place after 4 weeks of tDCS at home. Additionally, a researcher trained in the treatment, who was available to address potential questions and assist the participants, remotely supervised the home-based tDCS daily. Participants were instructed to maintain all prescribed medications stable throughout the study period, and no changes in pharmacological treatment were permitted during follow-up.

### Outcomes

The primary outcome was pain intensity, measured using the Visual Analogue Scale (0 = no pain, 10 = worst possible pain). Secondary outcomes included functional capacity, assessed by the Health Assessment Questionnaire–Disability Index (HAQ-DI), multidimensional pain-related disability, pressure pain threshold (PPT), fatigue, analgesic use, and treatment adherence.

### Instruments and assessment of outcomes

Outcomes were conducted at baseline (day 0), day 10, day 30 (end of treatment), and day 90 (follow-up) for the two primary outcomes: pain intensity and physical function. All other outcomes—Pressure Pain Threshold (PPT), Functional Assessment of Chronic Illness Therapy–Fatigue Scale (FACIT-F), and Brazilian Profile of Chronic Pain: Screen (B-PCP)—were assessed at baseline and at the end of treatment (day 30).

### Primary outcome

- *Pain intensity (VAS-pain)* was measured using the Visual Analogue Scale for Pain (VAS-pain), ranging from 0 (no pain) to 100 (maximum imaginable pain). Participants answered the question: “*How severe is your pain?*”

### Secondary outcomes

b. *Physical function (Health Assessment Questionnaire–Disability Index, HAQ-DI)* with 20-item instrument was used to assess functional capacity across eight domains of daily activity. Participants rated the level of difficulty from 0 (no difficulty) to 3 (unable to do). The final score corresponds to the mean of the eight categories: 0–1 = mild difficulty, 1–2 = moderate to severe difficulty 2–3 = severe disability [22].
c. Pain-related disability and emotional burden were assessed using the *Brazilian Profile of Chronic Pain: Screen (B-PCP)*, a multidimensional self-administered instrument that evaluates pain intensity, functional limitation, and emotional distress. Total scores range from 0 to 93, with higher values indicating greater pain-related severity and disability [23].
d. The Pressure Pain Threshold (PPT) was measured on the dominant forearm using an electronic algometer (J-Tech Medical Industries, Utah, USA). Participants were instructed to distinguish between pressure and pain and to verbally indicate when pain began. Three measurements were taken at 3–5-minute intervals, and the mean value was recorded.
e. The fatigue was assessed by the Functional Assessment of Chronic Illness Therapy–Fatigue Scale, FACIT-F) with 13-item, self-administered questionnaire. Participants rated their feelings over the past week on a scale from 0 (not at all) to 4 (very much). Lower scores indicate greater fatigue and worse quality of life [24].
f. Analgesic use was permitted as needed and were instructed to record their use in personal diaries. The total weekly dose taken during the treatment phase and the three-month follow-up period was used for analysis. Analgesic use was recorded in five frequency categories: *every day, three times per week, twice per week, once per week, and almost never*. For statistical analysis, these data were dichotomized into two groups: (1) frequent use (*every day or three times per week*), and (2) infrequent use (twice per week or less).
g. *Adherence* was evaluated based on the frequency and duration of home-based tDCS sessions, automatically recorded by the device and stored by the hospital’s Biomedical Engineering Service [21]. These data were downloaded and at the end of treatment.

### Assessment of clinical features, depressive symptoms, central sensitization, sleep quality and safety

h. Demographic information, medical comorbidities, diagnoses, fibromyalgia, medication use, medical procedures, and daily activities or emotions affected by worsening pain were obtained from the participants’ electronic medical records.
i. *Disease activity* was evaluated using the 28-joint Disease Activity Score (DAS-28), which assesses 28 joints, the participant’s global assessment (VAS, 0–100 mm), and inflammatory markers such as C-reactive protein (CRP): remission (<2.6), low (≥2.6 to <3.2) moderate (≥3.2 to ≤5.1), or high (DAS>5.1) [25].
j. *Central sensitization* was assessed using the Central Sensitization Inventory for the Brazilian population (CSI-BP), a 25-item self-administered questionnaire with a total score of 0–100. Scores of 0–29 indicate subclinical sensitization, 30–39 mild, 40–49 moderate, 50–59 severe, and 60–100 extreme sensitization [26].
k. *Adverse effects* were assessed using a structured questionnaire after 10 and 20 sessions. Participants rated severity as mild, moderate, or severe and could report additional effects through open-ended questions [27].
l. *Serum biomarkers* included concentrations of brain-derived neurotrophic factor (BDNF), tumor necrosis factor-α (TNF-α), interleukin-1 (IL-1), and interleukin-6 (IL-6) were determined using enzyme-linked immunosorbent assay (ELISA) kits (Elabscience, Wuhan, China). A standard curve was generated from the absorbance values of known concentrations provided in the kit. Biomarker concentrations in serum samples were interpolated from this curve and expressed in pg/mL [28].

### Sample size

The sample size was estimated based on the primary outcome, pain intensity (VAS-pain, 0–10). Calculations were performed using the PSS Health tool (version 32; https://hcpa-unidade-bioestatistica.shinyapps.io/PSS_Health/*)*, assuming a two-tailed α = 0.05 and 80% statistical power. A minimum clinically important difference (MCID) of 1.1 points on the VAS-pain was assumed between the a-tDCS and s-tDCS groups at the end of treatment (day 30), with a standard deviation (σ) = 2.0 [29]. Based on these parameters, a minimum of 16 participants per group was required. Considering a 15% dropout rate, a total sample of 34 participants (17 per group) was planned [30, 31]. Both estimations indicated a requirement of approximately 16 participants per group. Considering a 15% dropout rate, 34 participants (17 per group) was planned.

### Statistical analysis

Continuous and categorical variables were compared using Fisher’s exact test, the chi-square test, and the t-test for independent samples. The Shapiro-Wilk normality test was applied to determine the normal distribution of continuous variables. A Linear Mixed Model (LMM) for repeated measures assessed primary outcomes (pain severity and disability) with treatment, time, and the treatment-by-time interaction as fixed effects and included a random intercept for patients to account for time differences. The Bonferroni test adjustment was applied for post hoc multiple comparisons to identify differences between groups at each time point, and paired t-tests were used to assess within-group effects. A Generalized Linear Model (GLM) was employed to examine the treatment effects on the secondary outcomes, including heat pain threshold (HPP), Health Assessment Questionnaire–Disability Index (HAQ-DI), and Brazilian Profile of Chronic Pain: Screen (B-PCP). The analysis was planned according to the intention-to-treat (ITT) principle; however, since there were no dropouts or missing data, the analyses were performed per protocol. An exploratory general linear model (GLM) was performed to evaluate the effect of tDCS on pain intensity (ΔVAS), adjusted for biological and clinical factors. The models presented in Table 4 examined the effect of tDCS on pain intensity (ΔVAS). Model 4 A included baseline predictors (BDNF, CSI, and DMARD), whereas Model 4B incorporated within-subject changes in ΔBDNF and ΔCSI, with fibromyalgia diagnosis entered as a covariate. Interaction terms with treatment were explored, and effect sizes were estimated according to Cohen’s guidelines. All statistical analyses were two-tailed and conducted at a 5% significance level using SPSS Statistics, version 22.0 (IBM Corp., Chicago, IL, USA).

## Results

### Clinical, functional, and serum biomarker changes

**Fig. 2:**
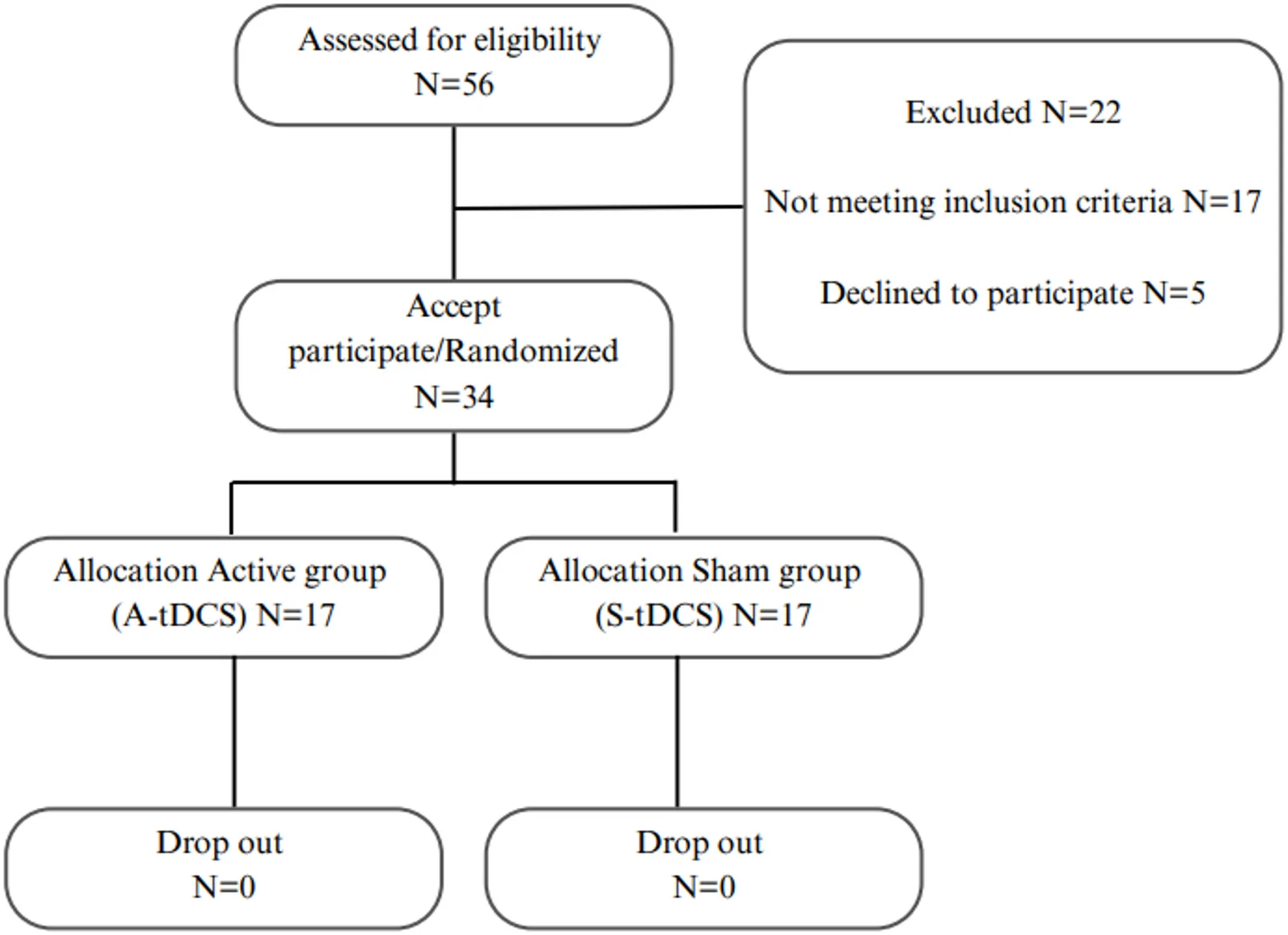
Consort flow diagram

Of the 56 patients screened, 34 were eligible for inclusion. Seventeen patients were excluded for not meeting the inclusion criteria, and five declined to participate. As a result, 34 patients were randomized to receive either anodal tDCS (a-tDCS; *n* = 17) or sham tDCS (*n* = 17) (Fig. 2). No participants discontinued the intervention or were lost to follow-up.

All participants (100%) in both groups reported the use of additional, non-prescribed NSAIDs for pain management. Table 1 summarizes the sociodemographic and clinical characteristics, showing no significant baseline differences between groups in age, sex, disease duration, or clinical parameters. Measures of disease activity, depressive symptoms, central sensitization, and sleep quality were comparable between groups, confirming adequate randomization and baseline homogeneity. Median CRP levels were 5.00 (1.0–10.0) mg/L in the a-tDCS group and 2.00 (1.0–9.0) mg/L in the sham group (*p* = 0.339). Disease activity was low in both groups, with a swollen joint count ≤ 1. Baseline pharmacological data included intermittent NSAID use reported by participants, as well as prescribed medications such as antidepressants and sleep agents (Table 1).

**Table 1:** Sociodemographic and clinical characteristics by treatment group 1 (s-tDCS or a-tDCS) (*n* = 34)

Variable	A-tDCS (*n* = 17)	S-tDCS (*n* = 17)	*p*
Age (years old), mean ± SD	57.23 ± 7.91	54.23 ± 9.53	0.32
Duration of RA (years), median (IQR)	9.00 (2–33)	11.00 (2–33)	0.77
Smoker, n (%)	0 (0)	2 (11.8)	0.16
csDMARDs, n (%)	12 (70.6)	15 (88.2)	0.21
bDMARDs, n (%)	2 (11.8)	3 (17.3)	0.64
JAK inhibitor, n (%)	0 (0)	0 (0)	0.99
Corticosteroid, n (%)	5 (29.4)	5 (29.4)	0.99
Antidepressant, n (%)	9 (52.9)	10 (58.8)	0.73
Non-steroidal anti-inflammatory drugs baseline, n (%)	17 (100)	17 (100)	0.99
Sleeping medicine baseline, n (%)	9 (52.9)	15 (88.2)	0.08
Formal education (ys)			0.87
Basic education, n (%)	7 (25)	7 (25)	-
High school, n (%)	8 (47.0)	5 (29.4)	-
College, n (%)	2 (11.7)	5 (29.4)	-
Professional status			0.26
Working, n (%)	4 (23.5)	7 (41.2)	-
Unemployed, n (%)	1 (5.9)	1 (5.9)	-
Retiree, n (%)	8 (47.1)	5 (29.4)	-
Government benefit (disability retirement), n (%)	3 (17.6)	4 (23.5)	-
Disease activity, depressive symptoms, central sensitization, sleep quality			
28-joint disease activity score (DAS-28)			
Central Sensitization Inventory	57.67 (14.18)	59.00 (12.91)	0.65
Diagnosis of fibromyalgia Yes/ No	9 (52.9%)/ 8 (47.1%)	12 (70.6%)/5 (29.4%)	0.24

csDMARD(conventional synthetic disease-modifying antirheumatic drugs): methotrexate, leflunomide, hydroxychloroquine, and sulfasalazine; bDMARDs (biologic disease-modifying antirheumatic drugs): adalimumab, etanercept, infliximab, certolizumab, golimumab, rituximab, tocilizumab, abatacept; n, number; SD, standard deviation; IQR, Interquartile; %, percent; Government benefit in Brazil correspond to individuals receiving disability-related benefits due to work incapacity

### Primary outcomes

#### Effect of treatment on pain scores (VAS)

**Fig. 3:**
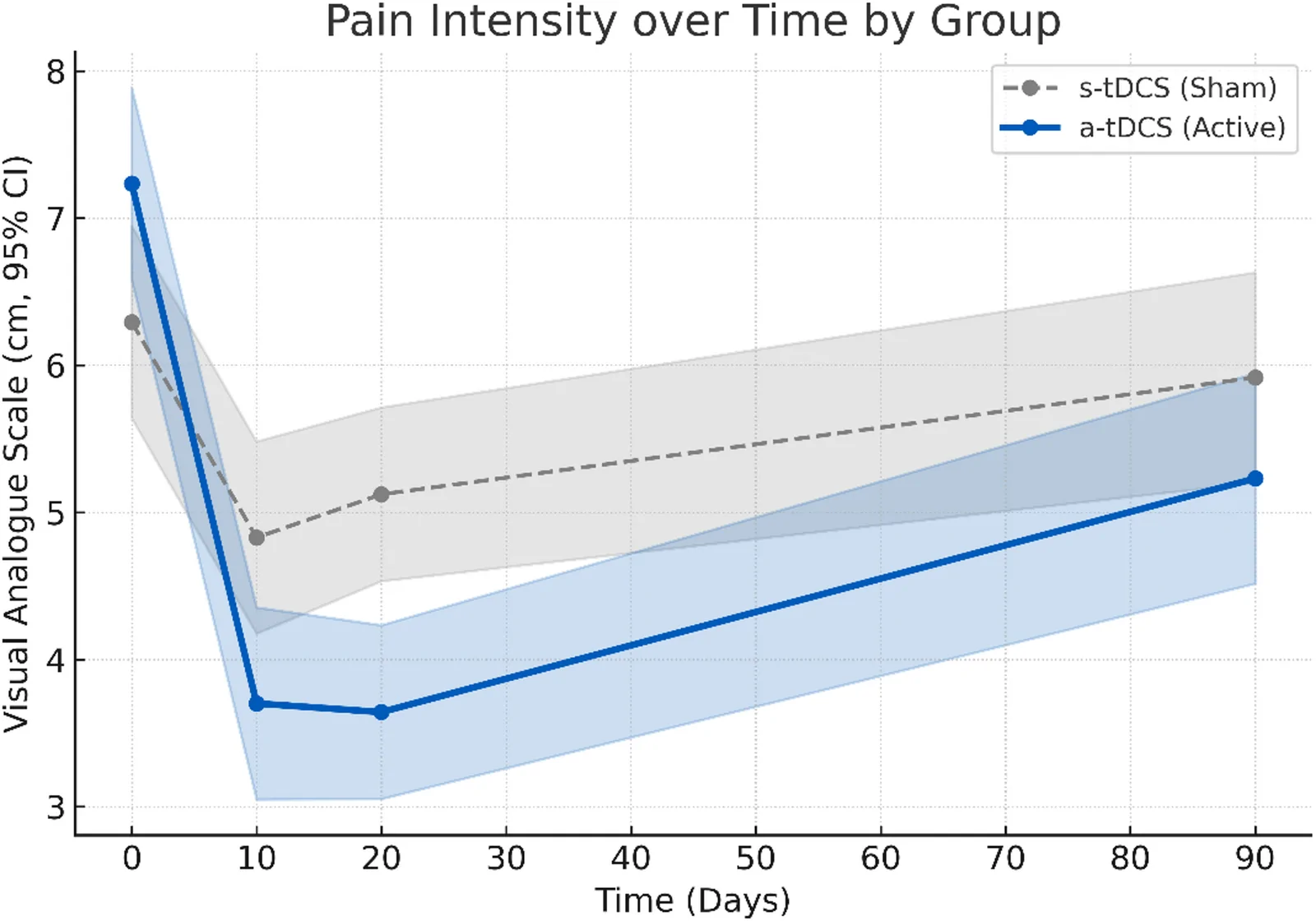
A linear mixed-effects model assessed the effect of treatment—anodal transcranial direct current stimulation (a-tDCS) versus sham tDCS—on VAS scores, adjusted for baseline pain. Solid lines depict mean values at each time point (Days 0, 10,30, and 90) for the a-tDCS group, and dashed lines represent the sham group. Shaded areas show 95% confidence intervals (CIs). Overlapping CIs indicate no statistically significant difference between groups

After treatment, the a-tDCS group showed a greater reduction in VAS-pain compared to the s-tDCS group (-33.53 mm vs. -14.12 mm, between-group difference of -19.41 mm; 95% CI, -29.29 to -9.52; *p* = 0.003) (Table 2). GLMM analysis showed a significant reduction in pain in the**a**-tDCS group, from 7.24 (1.27) at baseline to 5.23 (1.45) at 90 days, representing a 27.7% decrease from baseline (95% CI, − 42.8% to − 9.7%). In contrast, the sham group showed a smaller reduction, from 6.29 (1.36) to 5.92 (1.63), corresponding to a 6.0% decrease (Fig. 3). The between-group difference in percentage change was 21.7% (95% CI, 8.5%–34.9%), corresponding to a large effect size (Cohen’s d = 1.15).

**Fig. 4:**
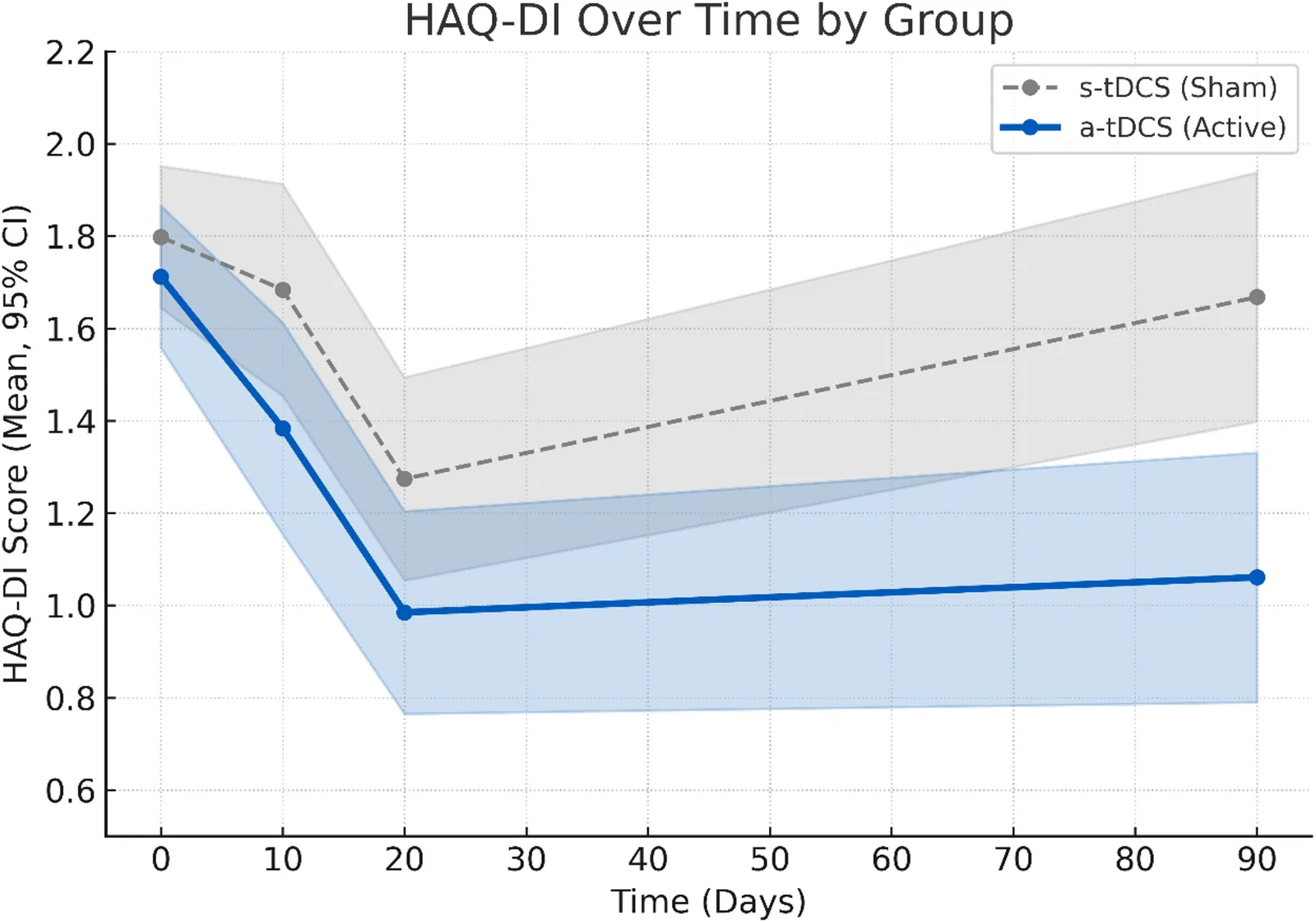
A linear mixed-effects model was used to assess the effect of treatment—anodal transcranial direct current stimulation (a-tDCS) versus sham tDCS—on functional scores (HAQ-DI), adjusted for baseline values. Solid lines represent mean values at each assessment point (days 0, 30, and 90) in the a-tDCS group, while dashed lines correspond to the sham group. Shaded areas indicate 95% confidence intervals (CIs). Overlapping CIs suggest that differences between treatment groups were not statistically significant

The model revealed a significant main effect of treatment (*F* = 15.79, *p* < 0.001) and time (*F* = 9.38, *p* < 0.001), with no treatment-by-time interaction (*F* = 0.78, *p* = 0.46) (Fig. 4). Baseline pain intensity (VAS₀) significantly predicted subsequent pain ratings (β = 0.51, 95% CI 0.31–0.71, *p* < 0.001), indicating that patients with higher initial pain tended to maintain higher pain levels throughout follow-up.

**Table 2:** Change in primary and additional outcomes from baseline after 30 days of tDCS (*n* = 34)

Mean change (CI 95%)	a-tDCS (*n* = 17)		s-tDCS (*n* = 17)		Difference (CI 95%)	Δp	ES
Primary outcomes	Baseline	After 30 days	Baseline	After 30 days			
VAS, (mm)	72.35 (61.97 to 79.20)	-33.53 (-42.69 to -24.36)	62.94 (75.72 to 57.21)	-14.11 (-18.40 to -9.83)	-19.41 (-29.29 to -9.52)	0.003*	1.0
**Secondary outcomes**							
HAQ-DI, (score)	1.75 (1.53 to 2.16)	-0.72 (-1.00 to -0.44)	2.10 (1.38 to 2.06)	-0.52 (-0.69 to -0.34)	0.20 (-0.11 to 0.51)	0.199	0.5
PPT (Kg/cm2)	1.50 (1.21 to 2.29)	0.96 (0.60 to 1.33)	1.80 (1.72 to 3.13)	0.36 (-0.24 to 0.97)	-0.60 (-1.28 to 0.07)	0.003*	0.6
FACIT-F, (score)	2.15 (1.51 to 2.36)	-0.74 (-1.08 to -0.40)	2.46 (1.83 to 2.73)	-0.83 (-1.18 to -0.49)	-0.09 (-0.55 to 0.37)	0.694	1.0

Mean and median values are presented (CI 95%). Δ,value after tDCS-baseline; n, number; CI 95%, confidence interval of 95%; Δp between groups, *p* < 0,05*; ES, effect size: ≤0.2 small, ≥ 0.5 medium, ≥ 0.8 large

### Secondary outcomes

#### Health assessment questionnaire – disability index - (HAQ-DI)

GLMM analysis showed a significant reduction in disability (HAQ-DI) in the a-tDCS group, from 1.71 (SD = 0.31) at baseline to 1.06 (SD = 0.55) at 90 days, representing a 38.0% decrease from baseline. In contrast, the sham group showed a smaller reduction, from 1.80 (SD = 0.31) to 1.67 (SD = 0.55), corresponding to a 7.2% decrease (Fig. 4). The between-group difference in percentage change was a reduction in a-tDCS of 30.8% (95% CI, 12.5%–49.0%), corresponding to a large effect size (Cohen’s d = 1.10).

The model revealed a significant main effect of treatment (F = 16.63, *p* < 0.001) and time (F = 16.11, *p* < 0.001), with no treatment-by-time interaction (F = 2.04, *p* = 0.11) (Fig. 4). Higher baseline disability also predicted greater functional difficulty across follow-up, regardless of treatment group (β = 0.75, *SE* = 0.07, *t*(102.7) = 10.90, *p* < 0.001; 95% CI = 0.61–0.88).

A-tDCS reduced the frequency of analgesic use compared with sham-tDCS. During treatment, 76.5% of participants in the a-tDCS group used analgesics fewer than three times per week, versus 23.5% in the s-tDCS group. The difference was significant (χ² = 6.03, *p* = 0.014), representing a 62% risk reduction in frequent analgesic use with active stimulation (RR = 0.38; 95% CI 0.18–0.79).

Table 3 summarizes the secondary outcomes. The general linear model (GLM) at the 90-day revealed that treatment effects differed across variables. A significant increase in the heat pain threshold and a greater reduction in disability in daily activities due to pain (B-PCP total score) were observed in the a-tDCS group compared with the sham group, whereas fatigue did not differ significantly between groups.

**Table 3:** Effects of treatment on fatigue, pain pressure threshold, and disability in daily activities due to pain across groups. Data are expressed as mean (SD) from pre- to post-intervention, with corresponding mean differences (Δ-values) (*n* = 34)

	Mean (SD) before (B) treatment	Mean (SD) after (A) treatment	∆-value (A-B)	β (SEM) [95% CI]	Wald χ2	df	*P*	ES^¥^
**Secondary outcomes**								
**Functional Assessment of Chronic Illness Therapy – Fatigue Scale**								
s-tDCS (*n* = 17)	2.28 (0.87)	1.44 (0.96)	0.84 (1.30)	0.09 (0.22) [− 0.34 to 0.52]	0.16	1	0.88	–
a-tDCS (*n* = 17)	1.94 (0.83)	1.19 (0.76)	0.75 (1.13)	0^reference^	–	–	–	–
**Heat pain threshold**								
s-tDCS (*n* = 17)	2.08 (1.20)	2.43 (1.37)	0.35 (1.82)	−0.69 (0.20) [− 1.09 to − 0.30]	12.00	1	0.001	0.59
a-tDCS (*n* = 17)	1.82 (0.99)	2.76 (1.14)	0.94 (1.51)	0^reference^	–	–	–	–
**Brazilian Profile of Chronic Pain: Screen (B-PCP) total score**								
s-tDCS (*n* = 17)	74.82 (19.89)	50.70 (21.11)	−22.52 (12.58)	11.23 (4.92) [1.58 to 20.89]	5.19	1	0.02	0.78
a-tDCS (*n* = 17)	73.75 (20.58)	40.00 (25.53)	−33.76 (16.75)	0reference	–	–	–	–

¥The effect size (ES) was calculated following Cohen’s guidelines. Additionally, Cramér’s V was used as an effect size measure, with the following interpretation: Small (0.10), Medium (0.30), and Large (0.50). Effect size (ES) according to guidelines (Cohen) was calculated based on the change from baseline used as a reference. https://www.psychometrica.de/effect_size.html. The symbol ‘---’ indicates that the effect size (ES) was not calculated because there was no significant difference between treatment groups

Table 4 summarizes the serum markers of inflammatory activity and brain-derived neurotrophic factor (BDNF) before and after the intervention for each treatment group. Among the biomarkers analyzed, a significant difference was observed only for BDNF, which showed a marked increase in the a-tDCS group compared with the sham stimulation. No significant between-group differences were found for the inflammatory markers (TNF-α, IL-1, or IL-6).

**Table 4:** Serum markers of inflammatory activity and brain-derived neurotrophic factor (BDNF) in a-tDCS and s-tDCS groups (*n* = 34)

	Mean (SD) before (B) treatment	Mean (SD) after (A) treatment	∆-value (A-B)	*P*
**Brain Derivate neurotrophic factor (BDNF)**				
s-tDCS(*n* = 17)	1280.57 ± 655.6	1207.22 ± 596.4	-73.35	0.041
a-tDCS (*n* = 17)	1289.88 ± 398.7	1446.52 ± 511.5	156.64	
**Tumor Necrosis Factor-alpha (TNF-α)**				
s-tDCS(*n* = 17)	19.81 ± 24.1	23.08 ± 33.3	3.27	0.220
a-tDCS (*n* = 17)	17.16 ± 19.0	23.14 ± 24.7	5.98	
**Interleukin-6 (IL-6)**				
s-tDCS(*n* = 17)	32.59 ± 34.6	26.84 ± 22.5	-5.75	0.487
a-tDCS (*n* = 17)	36.22 ± 31.6	32.97 ± 23.5	-3.25	
**Interleukin-6 (IL-6)**				
s-tDCS(*n* = 17)	0.38 ± 0.5	0.98 ± 0.9	0.6	0.326
a-tDCS (*n* = 17)	1.21 ± 1.9	1.56 ± 2.6	0.35	

Biomarkers in pg/ml; Meanvalues are presented (CI 95%). Δ,value after tDCS-baseline; n, number; CI 95%, confidence interval of 95%; Δp between groups, *p* < 0,05*

### Treatment effects and exploratory analysis of predictors of pain reduction

Table 4A summarizes the general linear model (GLM) evaluating predictors of change in pain intensity (ΔVAS) at the end of treatment. Active tDCS (a-tDCS) was significantly associated with a greater reduction in pain compared with sham stimulation. Higher baseline BDNF concentrations and lower CSI scores were independently associated with larger pain reductions. Moreover, a significant interaction between treatment and BDNF revealed that the analgesic effect of a-tDCS increased proportionally with higher BDNF levels, supporting a modulatory role of neurotrophic activity in treatment responsiveness. In contrast, the use of DMARDs seemed to mitigate the magnitude of pain improvement observed with a-tDCS.

Table 5B presents the GLM testing whether changes in pain intensity (ΔVAS) were predicted by ΔBDNF and ΔCSI, accounting for fibromyalgia diagnosis and treatment group. Active tDCS produced a significantly greater reduction in pain than sham. Larger increases in ΔBDNF and greater decreases in ΔCSI independently predicted stronger treatment effects. In patients with fibromyalgia, higher ΔBDNF potentiated the analgesic effect of active tDCS, supporting a neuroplastic contribution to treatment response. The three-way interaction including ΔCSI was not significant, indicating no additional modulation by changes in central sensitization. The results presented in Table 5A–B showed a treatment effect on pain intensity with a large effect size, both in models adjusted for baseline predictors and for within-subject changes from pre- to post-treatment.

**Table 5:** Predictors of pain reduction on the visual analogue scale (VAS) according to treatment group (a-tDCS vs. s-tDCS (*n* = 34)

**Model A –Predictors of tDCS Effects on Change in Pain Scores**					
**Pain intensity on Visual Analogue Scale (Δ-post minus pre scores)**					
	**Β (SEM) [95% CI]**	**Wald χ2**	**df**	**P**	**ES**
*Intercept*	-38.88 (12.08) [-62.55 to -15.20]	10.36	1	0.001	
s-tDCS (*n* = 17)	57.92 (14.61) [29.28 to 86.57]	15.71	1	0.000	0.68
a-tDCS(*n* = 17)	0^reference^				
Brain-derived neurotrophic factor (BDNF)	− 0.36 (0.15) [-0.65 to − 0.06]	5.68	1	0.017	0.41
Central Sensitization Inventory (CSI)	0.03 (0.009) [0.10 to 0.05]	9.06	1	0.003	0.57
Disease-Modifying Antirheumatic Drug (DMARD) (No/ Yes)	-13.86 (6.39) [-26.40 to -1.33]	4.69	1	0.030	0.37
*Interaction analysis*					
s-tDCS / a-tDCS at treatment end vs. BDNF	-0.03 (0.01) [-0.04 to − 0.0.008]	7.42	1	0.006	0.51
**Model B – Effects of tDCS on Pain Reduction according to (Δ-post-minus pre) BDNF and CSI**					
**Pain intensity on Visual Analogue Scale (Δ-post minus pre scores)**					
	**Β (SEM) [95% CI]**	**Wald χ2**	**df**	**P**	**ES**
*Intercept*	-21.05 (7.72) [-36.17 to -5.93]	-5.929	1	0.006	
s-tDCS (*n* = 17)	17.48 (4.87) [7.94 to 27.03]	27.030	1	0.000	0.62
a-tDCS(*n* = 17)	0^reference^	.	.	.	
(Δ) Brain-derived neurotrophic factor (BDNF)	0.04 (0.02) [0.006 to 0.08]	0.075	1	0.020	0.34
(Δ) Central Sensitization Inventory (CSI)	0.48 (0.22) [0.06 to 0.90]	0.903	1	0.026	0.37
Disease-Modifying Ant rheumatic Drug (DMARD) No/ Yes)	0.09 (5.27) [-10.24 to 10.41]	10.413	1	0.987	--
Diagnosis of fibromyalgia Yes/ No	1.91 (4.69) [-7.28 to 11.09]	11.097	1	0.684	---
**Interactions analysis: treatment vs. Δ: (post minus pre) BDNF vs. diagnosis of fibromyalgia**					
s-tDCS * (Δ: post minus pre) BDNF * fibromyalgia (No)	0.06 (0.06) [-0.08 to 0.15]	0.145	1	0.529	---
a-tDCS * (Δ: post minus pre) BDNF * fibromyalgia (Yes)	-0.07 (0.03) [-0.12 to -0.02]	-0.022	1	0.004	0.40
**Interactions analysis: treatment vs. Δ: (post minus pre) BDNF vs. diagnosis of fibromyalgia vs.**					
s-tDCS * (Δ: post minus pre) BDNF*fibromyalgia (No))* (Δ) CSI	0.003 (0.002) [-0.002 to 0.008]	0.008	1	0.233	--
a-tDCS * (Δ: post minus pre) BDNF*fibromyalgia (Yes)* (Δ) CSI	-0.001 (0.006) [-0.002 to 0.001]	0.000	1	0.081	--

¥The effect size (ES) was calculated following Cohen’s guidelines. Additionally, Cramér’s V was used as an effect size measure, with the following interpretation: Small (0.10), Medium (0.30), and Large (0.50). Effect size (ES) according to guidelines (Cohen) was calculated based on the change from baseline used as a reference. https://www.psychometrica.de/effect_size.html. The symbol ‘---’ indicates that the effect size (ES) was not calculated because there was no significant difference between treatment groups. Model B: ΔPain = b0 + b1*Group(active vs sham) + b2*ΔBDNF(post − pre) + b3*Fibromyalgia(No vs Yes) + b4*ΔCSI(post − pre) + b5*(Group × ΔBDNF) + b6*(Group×Fibromyalgia) + b7*(Group × ΔCSI) + error

### Assessment of adherence, adverse events and safety

Adherence to the protocol was determined by the number of completed sessions. Although 20 sessions were planned, 321 sessions (94.4%) were completed in the a-tDCS group, while participants in the s-tDCS group completed 299 of 340 sessions (88%), resulting in an overall adherence rate of 91.1% (620/680 sessions). The mean number of completed sessions was 18.88 ± 2.11 (94.4%) in the a-tDCS group and 17.64 ± 2.82 (88.2%) in the s-tDCS group, with no significant difference between groups (*P* = 0.16).

The most frequent adverse effects, considering all symptoms of any intensity (from mild to moderate), were sleepiness (a-tDCS = 41.2% vs. s-tDCS = 35.3%), headache (a-tDCS = 41.2% vs. s-tDCS = 35.3%), pain at the stimulation site (a-tDCS = 17.6% vs. s-tDCS = 41.1%), and burning sensation (a-tDCS = 17.6% vs. s-tDCS = 11.8%). All events were mild to moderate, with no severe adverse effects, and there were no significant differences between groups. Data area presented in Table 6.

**Table 6:** Side effects presented as percentage, and the incidence or severity of side effects classified as absence, mild, moderate, and severe (*n* = 34)

Symptoms	Group	Absence	Mild	Moderate	Severe	*P*-value
Headache	a-tDCS (*n* = 17)	10(58.8)	6 (35.3)	1 (5.9)	0 (0)	0.68
	s-tDCS (*n* = 17)	11 (64.7)	4 (23.5)	2 (11.8)	0 (0)	
Neck pain	a-tDCS (*n* = 17)	12 (70.6)	4 (23.5)	1 (5.9)	0 (0)	0.37
	s-tDCS (*n* = 17)	15 (88.2)	2 (11.8)	0 (0)	0 (0)	
Pain the area of stimulation	a-tDCS (*n* = 17)	14 (82.4)	2 (11.8)	1 (5.9)	0 (0)	0.19
	s-tDCS (*n* = 17)	10 (58.8)	7 (41.1)	0 (0)	0 (0)	
Itching	a-tDCS (*n* = 17)	14 (82.4)	2 (11.8)	1 (5.9)	0 (0)	0.10
	s-tDCS (*n* = 17)	12 (70.6)	2 (11.8)	0 (0)	0 (0)	
Burning	a-tDCS (*n* = 17)	14 (82.4)	3 (17.6)	0 (0)	0 (0)	0.34
	s-tDCS (*n* = 17)	15 (88.2)	2 (11.8)	0 (0)	0 (0)	
Redness	a-tDCS (*n* = 17)	16 (94.1)	1 (5.9)	0 (0)	0 (0)	0.50
	s-tDCS (*n* = 17)	17 (100)	0 (0)	0 (0)	0 (0)	
Sleepiness	a-tDCS (*n* = 17)	10 (58.8)	5 (29.4)	2 (11.8)	0 (0)	0.82
	s-tDCS (*n* = 17)	11 (64.7)	5 (29.4)	1 (5.9)	0 (0)	
Trouble of concentration	a-tDCS (*n* = 17)	17 (100)	0 (0)	0 (0)	0 (0)	0.99
	s-tDCS (*n* = 17)	17 (100)	0 (0)	0 (0)	0 (0)	

Data are n (%). An adverse event was present if the participant rated that it was at least remotely possible that it was associated with the intervention. Participants rated the severity of the adverse events as mild, moderate or severe. P values, determined with a two-sided Fisher’s exact test, represent the group differences of the total number of events per event category. *p* > 0.05*

## Discussion

This studysuggests that a four-week a-tDCS protocol applied over the primary motor cortex effectively reduced pain intensity and disability in patients with RA who remained symptomatic despite absence of objective findings of inflammation. The intervention was designed to explore the potential of home-based neuromodulation as a feasible, nonpharmacological strategy to address persistent pain and disability in RA, targeting central mechanisms that may maintain pain even in the absence of active inflammation. These findings reinforce the potential of tDCS as an accessible, non-pharmacological adjuvant therapy capable of modulating central mechanisms that sustain chronic pain in RA, even in the absence of active inflammation, thereby addressing the residual components of pain and functional impairment.

The present study suggests that twenty sessions (30 days of treatment) of home-based anodal tDCS applied over the primary motor cortex produced a significant reduction in pain intensity in patients with RA. Pain scores decreased from 7.24 ± 1.27 cm at baseline to 5.23 ± 1.45 cm after 90 days, representing a 27.7% reduction from baseline (95% CI, − 42.8% to − 9.7%). In contrast, the sham group exhibited only a minor improvement, from 6.29 ± 1.36 cm to 5.92 ± 1.63 cm, corresponding to a 6.0% decrease. The between-group difference in percentage change reached 21.7% (95% CI, 8.5%–34.9%), indicating a large effect size (Cohen’s d = 1.15). These findings confirm a robust analgesic effect of home-based a-tDCS, consistent with previous reports in fibromyalgia and other chronic pain conditions [32–35]. As shown in studies, stimulation of the primary motor cortex may reduce maladaptive connectivity and oscillatory activity within cortical–subcortical pain networks, engaging thalamic, limbic, and brainstem structures to enhance descending inhibitory control and pain modulation [36, 37].

Although participants receiving sham stimulation also reported modest pain relief, this change was likely driven by expectancy and contextual factors rather than sustained neuromodulatory effects. The small but measurable improvement in the sham group resembles findings from Khedr et al. [32], who reported minor VAS reductions after sham M1 stimulation in fibromyalgia. Minimal current exposure during sham sessions may transiently activate cortical regions, eliciting short-lived physiological responses [38]. Additionally, placebo-related mechanisms, such as heightened expectation of benefit and therapeutic engagement, can modulate pain perception through cognitive–affective pathways [34, 35, 38]. Klinger et al. [39] demonstrated that placebo conditioning effectively attenuates experimental pain, particularly when reinforced by expectations of clinical improvement, which may have contributed to the modest sham response observed in our study.

In line with our hypothesis, the magnitude of the analgesic effect observed in the active group a 27.7% reduction in pain intensity and a 38.0% improvement in functional capacity, represents a clinically meaningful outcome consistent with prior evidence supporting motor cortex stimulation as a neuromodulatory strategy for chronic pain [32–35]. Together, these results contribute to the evolving understanding of residual components of pain and functional impairment in rheumatoid arthritis and highlight the importance of integrating central modulation strategies into comprehensive disease management [32, 37, 40].

Active tDCS also enhanced pain pressure thresholds and improved a multidimensional pain index encompassing intensity, functional interference, and emotional distress, while significantly reducing the frequency of analgesic use throughout the treatment period. Similar multidimensional benefits have been reported in previous studies investigating motor cortex stimulation in chronic pain syndromes, which collectively support the involvement of this cortical region in both the sensory-discriminative and affective-emotional components of pain [37, 41]. In our study, the 62% reduction in analgesic use observed in the active group further reinforces previous clinical findings suggesting that tDCS can reduce pharmacological dependence and enhance endogenous pain modulation [38, 39, 42].

In studies exploring biological moderators of neuromodulation, higher baseline levels of BDNF have been associated with greater responsiveness to cortical stimulation [42, 43]. Consistent with these findings, patients in our sample with elevated BDNF and lower central sensitization scores showed greater pain reduction after active a-tDCS. This suggests that preserved neuroplastic potential reflected by higher BDNF availability may facilitate adaptive cortical reorganization and enhance treatment efficacy. Conversely, higher central sensitization seems to hinder tDCS effects, as described in other chronic pain populations, possibly due to entrenched maladaptive nociceptive processing that limits cortical flexibility [44].

The observed interaction between pharmacological and neuromodulatory factors also merits discussion. In accordance with reports from experimental and clinical studies, the DMARDs appeared to attenuate the magnitude of pain improvement induced by a-tDCS. This aligns with evidence that anti-inflammatory agents may alter neuroimmune signaling and interfere with plasticity-related molecular cascades [45]. It is plausible that DMARD-induced reductions in microglial activation and cytokine release modulate the responsiveness of cortical networks to external stimulation. In addition, exploratory analyses revealed that within-subject variations in serum BDNF and central sensitization indices may further modulate the clinical response to a-tDCS. Consistent with findings from other neuromodulation studies, increases in circulating BDNF were positively associated with pain reduction and functional improvement, supporting the hypothesis that tDCS may activate endogenous mechanisms of synaptic remodeling and cortical adaptation [46]. Likewise, reductions in central sensitization scores paralleled increases in pain thresholds, echoing prior reports that tDCS can recalibrate sensory gain and dampen central amplification of nociceptive signals [47]. Together, these findings suggest a dynamic interplay between biological plasticity and pain modulation during treatment, reinforcing the potential of BDNF and central sensitization as biomarkers of therapeutic responsiveness. In addition, baseline differences in TNF and interleukin levels should be interpreted cautiously. Although TNF levels were numerically higher and interleukin levels lower in the sham group, patients were clinically comparable according to DAS28 criteria. Circulating cytokine levels in rheumatoid arthritis may fluctuate and do not always parallel composite measures of disease activity [48].

RA patients experiencing chronic pain, even when their disease activity is under control, commonly report adopting a sedentary lifestyle, diminished motivation for daily activities, persistent fatigue, generalized weakness, depression and non-restorative sleep [46]. These reported incapacities adversely affect patients’ quality of life, social interactions, and work productivity [47, 49]. The baseline findings of our investigation validate these observations. After treatment, both groups demonstrated improvements in central sensitization, disability, fatigue and sleep quality. It is plausible that the observed effectiveness of tDCS on quality of life, fatigue, and sleep may stem from its beneficial impact on mood and depressive symptoms [35, 49].

The most common adverse effects were mild headache, neck pain, and sleepiness, all of short duration. Brunoni et al. (2011) [27] note that side effects like mild headache, itching, or burning are common but transient and harmless [27, 50]. In this study, there were no dropouts or exclusions. The A-tDCS group had 94% adherence to home treatment, completing 97% of the prescribed stimulation time, while the S-tDCS group achieved 88%. High adherence likely reflects tDCS’s safety, portability, affordability, and ease of use compared to other neuromodulation methods [27, 50]. Robust patient rapport and prompt responses to device-related concerns also had an influence in these results.

### Limitations

Despite the valuable insights gained from this study, some potential limitations must be acknowledged, such as the relatively small sample size and the duration of follow-up; subsequent assessments are required to ascertain the sustained effects of tDCS on pain parameters. In addition, large effect sizes derived from relatively small samples may overestimate the magnitude of treatment effects, reinforcing the need for replication in larger cohorts. A future multicenter trial would be particularly important to confirm the robustness and external validity of these findings. Furthermore, incorporating more objective measures such as functional brain imaging could facilitate the detection of subtle changes within the motor cortex that may be related to the observed effects.

It is also important to note that the sample consisted exclusively of right-handed women with low objective inflammatory findings and persistent pain, recruited from a single center. Therefore, caution is warranted when generalizing these results to men or to patients with moderate or high inflammatory disease activity. Nevertheless, given the relatively small sample size, the potential influence of baseline variability should be acknowledged when interpreting the findings. Larger studies will be important to confirm the stability and reproducibility of these results.

## Conclusion

The study provides novel and rigorous evidence that home-based anodal tDCS represents a safe, feasible, and effective adjunctive strategy for managing persistent pain in patients with rheumatoid arthritis who remain symptomatic despite controlled inflammation. The intervention produced clinically meaningful reductions in pain intensity and disability, accompanied by improvements in pain modulation and decreased analgesic use. Moreover, the modulation of biological markers and central sensitization supports the notion that tDCS engages neuroplastic mechanisms underlying pain chronification. Taken together, these findings highlight the potential of home-based neuromodulation as a practical and biologically grounded approach to complement conventional pharmacological treatment in rheumatoid arthritis.

## Supplementary Information

Below is the link to the electronic supplementary material.

Supplementary Material 1

## Data Availability

The datasets supporting the conclusions of this article were generated as part of a doctoral thesis conducted at the Hospital de Clínicas de Porto Alegre (HCPA) and the Federal University of Rio Grande do Sul (UFRGS). Due to ethical constraints and data protection regulations, the data are not publicly available but may be obtained from the corresponding author upon reasonable request and subject to institutional and ethics committee approval.

## References

[1] Lee YC, Nassikas NJ, Clauw DJ. The role of the central nervous system in the generation and maintenance of chronic pain in rheumatoid arthritis, osteoarthritis and fibromyalgia. Arthritis Res Ther. 2011;13(2):211. 21542893 10.1186/ar3306 PMC3132050

[2] Ferreira R, Pereira MG, Duarte C, et al. Pain and fatigue in rheumatoid arthritis patients with controlled disease: a systematic review. Rheumatol Int. 2021;41(2):257–69.33386447

[3] Altawil R, Saevarsdottir S, Wedrén S, Alfredsson L, Klareskog L, Lampa J. Remaining pain in early rheumatoid arthritis patients treated with methotrexate. Arthritis Care Res (Hoboken). 2016;68:1061–8. 26784398 10.1002/acr.22790 PMC5129578

[4] Vergne-Salle P, Pouplin S, Trouvin AP, Bera-Louville A, Soubrier M, Richez C, et al. The burden of pain in rheumatoid arthritis: impact of disease activity and psychological factors. Eur J Pain. 2020;24:1979–89. 32841455 10.1002/ejp.1651 PMC7692940

[5] Smith BH, et al. Central sensitization, chronic pain, and other symptoms. Cleve Clin J Med. 2023;90(4):245–54. 37011956 10.3949/ccjm.90a.22019

[6] Zhang A, Lee YC. Mechanisms for Joint Pain in Rheumatoid Arthritis (RA): from Cytokines to Central Sensitization. Curr Osteoporos Rep. 2018;16:603–10. 10.1007/s11914-018-0473-5. 30128836 10.1007/s11914-018-0473-5 PMC6157001

[7] Teixeira PEP, Brietzke AP, dos Santos VS, et al. The analgesic effect of transcranial direct current stimulation in fibromyalgia: a systematic review, meta-analysis, and meta-regression of potential influencers of clinical effect. Neuromodulation: Technol Neural Interface. 2023;26(4):715–27. 10.1016/j.neurom.2022.10.044 PMC10203058 36435660

[8] Moshfeghinia R, Faghani J, Khazaie H, et al. The effects of transcranial direct-current stimulation (tDCS) on pain intensity of patients with fibromyalgia: a systematic review and meta-analysis. BMC Neurol. 2023;23:395. 37919664 10.1186/s12883-023-03445-7 PMC10621179

[9] Fregni F, El-Hagrassy MM, Pacheco-Barrios K, et al. Evidence-based guidelines and secondary meta-analysis for the use of transcranial direct current stimulation in neurological and psychiatric disorders. Int J Neuropsychopharmacol. 2021;24(4):256–313. 32710772 10.1093/ijnp/pyaa051 PMC8059493

[10] Nitsche MA, Paulus W. Excitability changes induced in the human motor cortex by weak transcranial direct current stimulation. J Physiol. 2000;527(3):633–9. 10990547 10.1111/j.1469-7793.2000.t01-1-00633.x PMC2270099

[11] Boggio PS, Zaghi S, Lopes M, Fregni F. Modulatory effects of anodal transcranial direct current stimulation on perception and pain thresholds in healthy volunteers. Eur J Neurol. 2007;14(9):1124–30. 18717717 10.1111/j.1468-1331.2008.02270.x

[12] Wang S, Du SH, Wang XQ, Lu JY. Mechanisms of transcranial direct current stimulation (tDCS) for pain in patients with fibromyalgia syndrome. Front Mol Neurosci. 2024;17:1269636. 10.3389/fnmol.2024.1269636. 38356687 10.3389/fnmol.2024.1269636 PMC10865494

[13] Serrano-Máñez A, et al. Feasibility and efficacy of home-based tDCS combined with exercise for fibromyalgia. J Pain Res. 2022;15:257–68.35140512

[14] Brietzke AP, et al. Home-based transcranial direct current stimulation for fibromyalgia: a randomized, double-blind, sham-controlled trial. Pain. 2020;161(8):1789–99.

[15] Hopewell S, Chan A, Collins GS, HrÃ³bjartsson A, Moher D, Schulz KF, et al. CONSORT 2025 statement: updated guideline for reporting randomised trials BMJ. 2025; 389:e081123 10.1136/bmj-2024-081123 10.1136/bmj-2024-081123 PMC11995449 40228833

[16] Daniel et al. Rheumatoid arthritis classification criteria: an American college of rheumatology/european league against rheumatism collaborative initiative aletaha. Annals of the Rheumatic Diseases. 2010;69(9): 1580–1588. 10.1136/ard.2010.138461 20699241

[17] Fregni F, Nitsche M, Loo CK, et al. Regulatory Considerations for the Clinical and Research Use of Transcranial Direct Current Stimulation (tDCS): review and recommendations from an expert panel. Clin Res Regul Aff. 2015;32(1):22–35. 10.3109/10601333.2015.980944. 25983531 10.3109/10601333.2015.980944 PMC4431691

[18] Caumo W, de Souza A, Vidor LP, et al. Efficacy of home-based transcranial direct current stimulation over the primary motor cortex and dorsolateral prefrontal cortex in the disability due to pain in fibromyalgia: a factorial sham-randomized clinical study. J Pain. 2024;25(4):376–92. 37689323 10.1016/j.jpain.2023.09.001

[19] Caumo W, et al. Predictors of response to home-based transcranial direct current stimulation in chronic pain: a longitudinal study. J Pain Res. 2025;18:1–13.39776764

[20] Carvalho F, Brietzke AP, Gasparin A, et al. Home-based transcranial direct current stimulation device development: an updated protocol used at home in healthy subjects and fibromyalgia patients. J Vis Exp. 2018;13757614. 10.3791/57614. 10.3791/57614 PMC6126460 30059026

[21] Pacheco-Barrios K, Cardenas-Rojas A, Thibaut A, Costa B, Ferreira I, Caumo W, et al. Methods and strategies of tDCS for the treatment of pain: current status and future directions. Expert Rev Med Devices. 2020;17:879–98. 10.1080/17434440.2020.1816168. 32845195 10.1080/17434440.2020.1816168 PMC7674241

[22] Bruce B. and JFFries. The Health Assessment Questionnaire (HAQ). Suppl 39: S14-8. Linical Experimental Rheumatol. 2005;23:5. 16273780

[23] Wolnei Caumo LS, Ruehlman P, Karoly. Francisléa Sehn, Liliane Pinto Vidor, Letizzia Dall-Ágnol, Mônica Chassot, Iraci L. S. Torres, Cross-Cultural adaptation and validation of the profile of chronic pain: screen for a Brazilian population, pain medicine. 2013;14(1): 52–61. 10.1111/j.1526-4637.2012.01528.x. 10.1111/j.1526-4637.2012.01528.x 23171145

[24] Cella DYSSMCESNGJ. Validation of the Functional Assessment of Chronic Illness Therapy Fatigue Scale relative to other instrumentation in patients with rheumatoid arthritis. J Rheumatol. 2005;32(5):811–9. PMID: 15868614. 2005. 15868614

[25] Anderson J, Caplan L, Yazdany J, Robbins ML, Neogi T, Michaud K, et al. Rheumatoid arthritis disease activity measures: American College of Rheumatology recommendations for use in clinical practice. Arthritis Care Res (Hoboken). 2012;64:640–7. 10.1002/ACR.21649. 22473918 10.1002/acr.21649 PMC4028066

[26] Caumo W, Antunes LC, Elkfury JL, Herbstrith EG, Sipmann RB, Souza A, et al. The Central Sensitization Inventory validated and adapted for a Brazilian population: psychometric properties and its relationship with brain-derived neurotrophic factor. J Pain Res. 2017;10:2109. 10.2147/JPR.S131479. 28979158 10.2147/JPR.S131479 PMC5589103

[27] Brunoni AR, Amadera J, Berbel B, Volz MS, Rizzerio BG, Fregni F. A systematic review on reporting and assessment of adverse effects associated with transcranial direct current stimulation. Int J Neuropsychopharmacol. 2011;14:1133–45. 10.1017/S1461145710001690. 21320389 10.1017/S1461145710001690

[28] Caumo W, Deitos A, Carvalho S, Leite J, Carvalho F, Dussán-Sarria JA, et al. Motor cortex excitability and BDNF Levels in chronic musculoskeletal pain according to structural pathology. Front Hum Neurosci. 2016;10. 10.3389/fnhum.2016.00357. 10.3389/fnhum.2016.00357 PMC4946131 27471458

[29] Dworkin RH, Turk DC, McDermott MP, Peirce-Sandner S, Burke LB, Cowan P, et al. Interpreting the clinical importance of treatment outcomes in chronic pain clinical trials: IMMPACT recommendations. Pain. 2008;137(2):248–52. 10.1016/j.pain.2008.02.034. 10.1016/j.jpain.2007.09.005 18055266

[30] Wells GA, Tugwell P, Kraag GR, Baker PR, Groh J, Redelmeier DA. Minimum important difference between patients with rheumatoid arthritis: the patient’s perspective. J Rheumatol. 1993;20(3):557–60. 8478873

[31] Kosinski M, Zhao SZ, Dedhiya S, Osterhaus JT, Ware JE Jr. Determining minimally important changes in generic and disease-specific health-related quality of life questionnaires in clinical trials of rheumatoid arthritis. Arthritis Rheum. 2000;43(7):1478–87. 10902749 10.1002/1529-0131(200007)43:7<1478::AID-ANR10>3.0.CO;2-M

[32] Khedr EM, Omran EAH, Ismail NM, El-Hammady DH, Goma SH, Kotb H, et al. Effects of transcranial direct current stimulation on pain, mood and serum endorphin level in the treatment of fibromyalgia: A double blinded, randomized clinical trial. Brain Stimul. 2017;10:893–901. 10.1016/j.brs.2017.06.006. 28684258 10.1016/j.brs.2017.06.006

[33] Gurdiel-Álvarez F, González-Zamorano Y, Lerma-Lara S, Gómez-Soriano J, Sánchez-González JL, Fernández-Carnero J, et al. Transcranial Direct Current Stimulation (tDCS) Effects on Quantitative Sensory Testing (QST) and Nociceptive Processing in Healthy Subjects: A Systematic Review and Meta-Analysis. Brain Sci. 2023;14:9. 10.3390/BRAINSCI14010009/S1. 38275514 10.3390/brainsci14010009 PMC10813344

[34] Boggio PS, Ferrucci R, Rigonatti SP, Covre P, Nitsche M, Pascual-Leone A, et al. Effects of transcranial direct current stimulation on working memory in patients with Parkinson’s disease. J Neurol Sci. 2006;249:31–8. 10.1016/j.jns.2006.05.062. 16843494 10.1016/j.jns.2006.05.062

[35] Ribeiro H, Sesterhenn RB, De Souza A, De Souza AC, Alves M, Machado JC, et al. Preoperative transcranial direct current stimulation: Exploration of a novel strategy to enhance neuroplasticity before surgery to control postoperative pain. A randomized sham-controlled study. PLoS ONE. 2017;12. 10.1371/JOURNAL.PONE.0187013. 10.1371/journal.pone.0187013 PMC5708693 29190741

[36] Polanía R, Paulus W, Antal A, Nitsche MA. Introducing graph theory to track for neuroplastic alterations in the resting human brain: A transcranial direct current stimulation study. NeuroImage. 2011;54:2287–96. 10.1016/j.neuroimage.2010.09.085. 20932916 10.1016/j.neuroimage.2010.09.085

[37] Boggio PS, Zaghi S, Lopes M, Fregni F. Modulatory effects of anodal transcranial direct current stimulation on perception and pain thresholds in healthy volunteers. Eur J Neurol. 2008;15:1124–30. 10.1111/j.1468-1331.2008.02270.x. 18717717 10.1111/j.1468-1331.2008.02270.x

[38] Benedetti F, Mayberg HS, Wager TD, Stohler CS, Zubieta J-K. Neurobiological Mechanisms of the Placebo Effect. J Neurosci. 2005;25:10390–402. 10.1523/JNEUROSCI.3458-05.2005. 16280578 10.1523/JNEUROSCI.3458-05.2005 PMC6725834

[39] Klinger R, Soost S, Flor H, Worm M. Classical conditioning and expectancy in placebo hypoalgesia: A randomized controlled study in patients with atopic dermatitis and persons with healthy skin. Pain. 2007;128:31–9. 10.1016/j.pain.2006.08.025. 17030095 10.1016/j.pain.2006.08.025

[40] Polanía R, Paulus W, Nitsche MA. Modulating cortico-striatal and thalamo‐cortical functional connectivity with transcranial direct current stimulation. Hum Brain Mapp. 2012;33:2499–508. 10.1002/hbm.21380. 21922602 10.1002/hbm.21380 PMC6870027

[41] Fregni F, Gimenes R, Valle AC, Ferreira MJL, Rocha RR, Natalle L, et al. A randomized, sham-controlled, proof of principle study of transcranial direct current stimulation for the treatment of pain in fibromyalgia. Arthritis Rheum. 2006;54:3988–98. 10.1002/art.22195. 17133529 10.1002/art.22195

[42] Chassot M, Dussan-Sarria JA, Sehn FC, Deitos A, de Souza A, Vercelino R, et al. Electroacupuncture analgesia is associated with increased serum brain-derived neurotrophic factor in chronic tension-type headache: a randomized, sham controlled, crossover trial. BMC Complement Altern Med. 2015;15:144. 10.1186/s12906-015-0664-x. 25947167 10.1186/s12906-015-0664-x PMC4429917

[43] Caumo W, Alves RL, Vicuña P, Alves CF da, Ramalho S, Sanches L. Impact of Bifrontal Home-Based Transcranial Direct Current Stimulation in Pain Catastrophizing and Disability due to Pain in Fibromyalgia: A Randomized, Double-Blind Sham-Controlled Study. J Pain. 2022;23:641–56. 10.1016/j.jpain.2021.11.002. 34785366 10.1016/j.jpain.2021.11.002

[44] van Hecke O, Torrance N, Smith BH. Chronic pain epidemiology and its clinical relevance. Br J Anaesth. 2013;111:13–8. 10.1093/bja/aet123. 23794640 10.1093/bja/aet123

[45] Lewis GN, Rice DA, McNair PJ. Conditioned Pain Modulation in Populations With Chronic Pain: A Systematic Review and Meta-Analysis. J Pain. 2012;13:936–44. 10.1016/j.jpain.2012.07.005. 22981090 10.1016/j.jpain.2012.07.005

[46] Loo CK, Alonzo A, Martin D, Mitchell PB, Galvez V, Sachdev P. Transcranial direct current stimulation for depression: 3-week, randomised, sham-controlled trial. Br J Psychiatry. 2012;200:52–9. 10.1192/bjp.bp.111.097634. 22215866 10.1192/bjp.bp.111.097634

[47] Hsu W-Y, Cheng C-H, Zanto TP, Gazzaley A, Bove RM. Effects of transcranial direct current stimulation on cognition, mood, pain, and fatigue in multiple sclerosis: a systematic review and meta-analysis. Front Neurol. 2021;12. 10.3389/fneur.2021.626113. 10.3389/fneur.2021.626113 PMC7982804 33763014

[48] McInnes IB, Schett G. The pathogenesis of rheumatoid arthritis. N Engl J Med. 2011;365(23):2205-19. 10.1056/NEJMra1004965. PMID: 22150039. 10.1056/NEJMra1004965 22150039

[49] Samartin-Veiga N, González-Villar AJ, Pidal-Miranda M, Vázquez-Millán A, Carrillo-de-la-Peña MT. Active and sham transcranial direct current stimulation (tDCS) improved quality of life in female patients with fibromyalgia. Qual Life Res. 2022;31:2519–34. 10.1007/s11136-022-03106-1. 35229253 10.1007/s11136-022-03106-1 PMC9250466

[50] Chase HW, Boudewyn MA, Carter CS, Phillips ML. Transcranial direct current stimulation: a roadmap for research, from mechanism of action to clinical implementation. Mol Psychiatry. 2020;25:397–407. 10.1038/s41380-019-0499-9. 31455860 10.1038/s41380-019-0499-9 PMC6981019

